# Influenza-associated paediatric respiratory hospitalizations in China, 1996–2012: a systematic analysis

**DOI:** 10.5365/wpsar.2018.9.1.004

**Published:** 2018-10-09

**Authors:** Mei Shang, Kathryn E. Lafond, Jeffrey McFarland, Suizan Zhou, John Klena, Marc-Alain Widdowson

**Affiliations:** a China-US Collaborative Program on Emerging and Reemerging Infectious Diseases, Centers for Disease Control and Prevention, Beijing, China.; b Influenza Division, Centers for Disease Control and Prevention, Atlanta, GA, USA.

## Abstract

**Background:**

The World Health Organization recommends that children aged ≥ 6 months be vaccinated against influenza. Influenza vaccination policies depend on the evidence of the burden of influenza, yet few national data on influenza-associated severe outcomes among children exist in China.

**Methods:**

We conducted a systematic review of articles published from 1996 to 2012 on laboratory-confirmed, influenza-associated paediatric respiratory hospitalizations in China. We extracted data and stratified the percentage of samples testing positive for influenza by age group (< 2, < 5 and < 18 years old); case definition; test methods; and geographic location. The pooled percentage of samples testing positive for influenza was estimated with a random effects regression model.

**Results:**

Influenza was associated with 8.8% of respiratory hospitalizations among children aged < 18 years, ranging from 7.0% (95% confidence interval: 4.2–9.8%) in children aged < 2 years to 8.9% (95% confidence interval: 6.8–11%) in children aged < 5 years. The percentage of samples testing positive for influenza was consistently higher among studies with data from children aged < 5 years and < 18 years than those restricted only to children aged < 2 years; the percentages were higher in Northern China than Southern China.

**Discussion:**

Influenza is an important cause of paediatric respiratory hospitalizations in China. Influenza vaccination of school-aged children could prevent substantial influenza-associated illness, including hospitalizations, in China.

Young children are at an increased risk of severe disease due to influenza infection compared to older children and young adults. (*1*-*5*) Data from temperate northern hemisphere countries indicate that rates of influenza-associated hospitalizations among children aged < 5 years range from 0.36 to 5.16 per 1000 children with the highest rate among children aged < 2 years. (*4*, *6*, *7*) Therefore, the World Health Organization (WHO) recommends the inclusion of children aged 6 to 59 months as a priority group for seasonal influenza vaccination. (*8*) WHO also provides global guidance on surveillance for influenza, including influenza-like illness (ILI) among outpatients and severe acute respiratory infection (SARI) among inpatients, to capture influenza epidemiology, including disease burden. (*9*)

Few nationally representative studies exist on influenza-associated severe disease among children in China. Since 2007, the Chinese Center for Disease Control and Prevention (China CDC) has recommended annual seasonal influenza vaccination for children aged ≥ 6 months (*10*, *11*) based largely on disease burden data from other northern hemisphere countries. However, influenza vaccine uptake among children remains low. A telephone survey in four provinces, representing eastern and central China, found that influenza vaccination coverage among children aged < 5 years in urban settings was 21.9% for the 2009–2010 season and 25.6% for the 2011–2012 season. (*12*)

To better understand the epidemiology of influenza and influenza-associated disease burden, China CDC has implemented national and provincial-level surveillance systems. ILI surveillance, which began in 2009, monitors the predominant influenza virus strains circulating in outpatient settings and covers all provinces in mainland China, but it is not designed to estimate the disease burden. In 2011, China CDC also began inpatient surveillance for SARI in 10 provinces; however, the surveillance only covers a limited geographic area, mostly in the more developed eastern provinces. Furthermore, SARI sentinel site surveillance uses a modified version of the WHO SARI case definition which includes different criteria for patients aged > 5 years and ≤ 5 years and is also more specific. (*13*) Therefore, SARI surveillance likely underestimates the impact of influenza-associated hospitalizations nationwide, particularly those that do not fall within the strict SARI case definition. The contribution of influenza among respiratory hospitalizations in children aged < 18 years remains a key knowledge gap.

To address this gap, we conducted a systematic review of the Chinese and English literature to assess the burden of influenza-associated paediatric respiratory hospitalizations in China. We wanted to better understand influenza-associated hospitalizations, especially during the period when relevant data were not well described. The evidence provided will help to improve estimates of the influenza burden in China, refine influenza vaccination policy and reduce nationwide influenza-associated paediatric morbidity and mortality.

## Methods

### Systematic review of the literature

We conducted a systematic search of biomedical reference databases (PubMed, Embase, Web of Science, CINAHL, IndMed, LILACS, WHOLIS, CNKI and Global Health) to identify articles published from 1 January 1996 to 31 December 2012. Keywords that were used for searching were grouped in two categories: respiratory infection and viral etiology (full list of search terms and results are provided in **Supplementary Table 1**). We adopted the same literature search strategy as the one used to assess influenza-associated paediatric respiratory hospitalizations at the global level. (*14*) Two independent reviewers screened the identified papers to select those that met the following inclusion criteria: (1) presented original data; (2) the study population included Chinese children aged < 18 years old; (3) collected data from a minimum of 12 months of continuous surveillance; (4) conducted laboratory testing for influenza; (5) stated a pre-specified case definition or other clear criteria for specimen collection and testing; (6) included hospitalized case-patients (nosocomial infections were excluded); (7) provided both the numerator and denominator for influenza testing; (8) and tested a minimum of 50 children for influenza infection. For papers meeting these criteria, the full-text articles were obtained and re-screened by two reviewers. Key data that were extracted included study duration in years; geographic location of the study (defined as Northern or Southern China; demarcated by the Qinling Mountains-Huaihe River line); (*15*) total numbers of inpatients tested and total numbers tested positive for influenza; age group; case definition used to screen patients for testing (e.g. community-acquired pneumonia, SARI and acute respiratory infection); type of diagnostic tests used; and publication year. Since distinct age groups in the published data were not standardized for easy comparison, we created three overlapping age groups: < 2 years, < 5 years and < 18 years old. The < 18 years old group included the < 2 years and < 5 years old groups, and the < 5 years old group included the < 2 years old group. To ensure the disease burden data are associated with seasonal influenza, we excluded results that covered the 2009 influenza A(H1N1) pandemic period.

### Quality assessment

Data quality for each eligible article was scored using a modified Newcastle-Ottawa checklist for bias assessment (*16*) with three standards: (1) representativeness of the sampling process for enrolment; (2) specificity of enrolment criteria; (3) and clarity of reported results. A score of one or zero was given to each item accordingly.

### Statistical analyses

We first described eligible studies by age group, study duration, total numbers tested and positive for influenza infection, case definition, type of diagnostic tests used and geographic location. We calculated the percentage of samples that tested positive for influenza (hereafter referred to as per cent influenza-positive). Kruskal–Wallis one-way analysis of variance was used to test the difference among medians. (*17*) We then calculated pooled estimates of the per cent influenza-positive using a Dersimonian and Laird random effects meta-analysis model (*18*) for the three age groups stratified by geographic location. Briefly, in the random effect model, we assumed that the per cent influenza-positive estimated in the different studies were not identical. Because of variations in the sensitivity and specificity of different diagnostic tests, (*19*-*27*) we also calculated pooled estimates stratified by a different diagnostic approach: (1) diagnosis based on polymerase chain reaction (PCR); (2) any diagnostic test except alkaline phosphatase-anti-alkaline phosphatase technique (APAAP); (3) any diagnostic test except immunoassay; (4) any diagnostic test except APAAP or immunoassay. All reported tests were two-sided, and *P*-values < 0.05 indicated statistical significance. Data were analysed using Stata, version 12 (StataCorp, College Station, TX).

## Results

### Study characteristics

**Figure 1:**
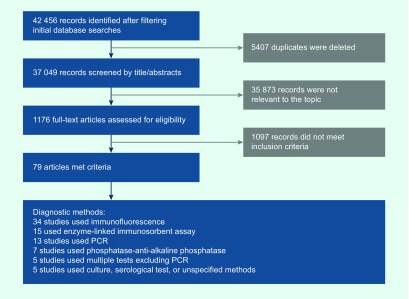
**Flow diagram for systematic review process**

**Figure 2:**
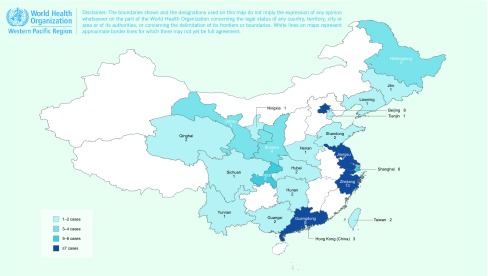
The distribution of studies included in the systematic analysis*

The systematic literature search identified 42 456 unique records (4450 Chinese and 38 006 English) from the nine scientific literature databases. A total of 1176 full-text articles (219 in Chinese and 957 in English) were reviewed. After excluding articles that did not meet the inclusion criteria and those with overlapping populations, 79 articles (69 in Chinese and 10 in English) were included in the descriptive analysis (**Fig. 1**). The full list of included articles is provided in **Supplementary Table 2**. The number of available studies published before 2004 was limited (*n* = 12). The number of studies increased to eight in 2004 and 14 in 2010, reflecting the 2009 influenza A(H1N1) pandemic. The data sets covered 23 provinces and special administrative regions (**Fig. 2**). Of the 79 articles included, 50 studies (63%) were from Southern China and 29 (37%) were from Northern China (**Table 1**). More than 95% of the studies tested at least 100 patients during the study period. More than 40% of the studies used immunofluorescence as the diagnostic test. Most studies differentiated influenza A and influenza B (*n* = 60 of 79). Over the years, influenza A positivity remained higher than influenza B positivity (median, interquartile range [IQR]: 2.5% [1.2–7.3%] vs 0.5% [0.2–4.1%]).

[**insert** Figure 1]

[**insert** Figure 2]

**Table 1:** Characteristics of published studies and data sources about influenza-associated paediatric respiratory hospitalizations in China, 1996–2012 (*n* = 79)

Characteristics	Number of published studies (%)
**Age group in years**	**-**
< 2	48 (61)
< 5	53 (67)
< 18	79 (100)
**Study duration in years**	**-**
1–2	61 (77)
3–4	14 (18)
≥ 5	4 (5)
Geographic location*	** -**
Northern China	50 (63)
Southern China	29 (37)
**Total cases tested**	** -**
0–99	2 (3)
100–499	30 (38)
500–999	13 (16)
≥ 1 000	34 (43)
**Diagnostic test**	** -**
Polymerase chain reaction (PCR) only	10 (13)
Immunofluorescence only	34 (44)
Multiple diagnostic tests, incl. PCR	3 (4)
Multiple diagnostic tests, excl. PCR	5 (6)
ELISA^#^	15 (20)
APAAP^†^	7 (9)
Others^‡^	4 (5)
**Case definition**	** -**
Acute respiratory infection	32 (41)
Acute lower respiratory infection	23 (29)
Pneumonia	17 (22)
**Others^§^**	**7 (9)**

* Southern and Northern are defined by national standards, which is Qing Mountain and Huai River line. (*15*)

^†^ APAAP: Alkaline phosphatase-anti-alkaline phosphatase technique.

^#^ ELISA: Enzyme-linked immunosorbent assay

^‡^ Others included pneumonia & bronchiolitis (***n*** = 1), SARI (***n*** = 1), bronchiolitis (***n*** = 2) and others (***n*** = 3).

^§^ Others included culture (***n*** = 1), serological test (***n*** = 1), non-classified (***n*** = 2).

The most commonly used case definitions for screening were acute respiratory infection (ARI), acute lower respiratory infection and pneumonia. ARI case definitions varied in different settings, but mostly met one or more of the following criteria: (1) symptoms of acute infection; (2) a body temperature > 38.0 °C; (3) white blood cell count of > 10 000/ml; (4) and signs/symptoms of acute respiratory illness. Only one study used SARI as a case definition. For patients > 5 years, SARI is defined as an acute onset of elevated temperature (axillary temperature ≥ 38 °C), cough or sore throat tachypnea (respiratory rate ≥ 25/min) or dyspnea (difficulty breathing) either at admission or during stay. For patients aged ≤ 5 years, SARI is defined as an acute onset of cough or dyspnea either at admission or during stay, and at least one of the following six signs or symptoms: (1) tachypnea (respiratory rate > 60/min for those aged < 2 months, respiratory rate > 50/min for those aged 2 to < 12 months, respiratory rate > 50/min for those aged 1 to ≤ 5 years); (2) inability to drink or breastfeed; (3) vomiting; (4) convulsions; (5) lethargy or unconsciousness; (6) and chest in-drawing or stridor in a calm child.

### Crude median per cent influenza-positive

The crude median per cent influenza-positive among studies with data from children aged < 2 years was 2% (IQR: 1–8%) and from children aged < 5 years and < 18 years was 6% (IQR: 2–11%, **Table 2**). The crude median per cent influenza-positive was four times lower among the 34 data sets that used immunofluorescence alone as compared to the 44 data sets that used other methods (2% versus 8%, Kruskal–Wallis test *P* < 0.05). The crude per cent influenza-positive was almost four times higher among the seven data sets that used APAAP as compared to the 71 data sets that used other methods (19% versus 5%; Kruskal–Wallis test *P* < 0.05). Stratification by age did not change the patterns. The crude per cent influenza-positive was not associated with case definition, geographic location or study duration (Kruskal–Wallis test *P* > 0.05 for all).

**Table 2:** Crude proportion of respiratory samples from hospitalized children testing positive for influenza by age group, diagnostic test, case definition, clinical diagnosis and geographic location in China, 1996–2012

Characteristic	No. studies (*n* = 79)	No. tested, Median (IQR)	No. positive, Median (IQR)	Median percentage influenza-positive samples (IQR)	*P*-value
**Age group in years**	** -**	**-**	**-**	**-**	**-**
< 2	48	978 (320–2143)	48 (17–99)	2 (1–8)	^‡^
< 5	53	821 (302–2073)	41 (17–85)	6 (2–11)	-
< 18	79	796 (280–1908)	39 (15–80)	6 (2–11)	-
**Diagnostic test**	**-**	**-**	**-**	**-**	**0.09^§^**
PCR	10	482 (340–961)	41 (35–65)	8 (7–12)	-
Immunofluorescence only	34	1216 (412–2646)	30 (14–80)	2 (2–6)	-
Multiple diagnostic tests including PCR	3	120 (116–469)	12(6–53)	10 (5–11)	-
Multiple diagnostic tests excluding PCR	5	1022 (672–1031)	44 (27–85)	6 (2–8)	-
ELISA	15	353 (144–837)	25 (7–70)	8 (2–16)	-
APAAP	7	1216 (169–5328)	113 (25–494)	19 (8–28)	-
Others*	4	1801 (856–7136)	96 (46–180)	6 (3–8)	-
**Case definition**	**-**	**-**	**-**	**-**	**-**
Acute respiratory infection	32	1027 (258–2667)	45 (16–116)	3 (2–8)	0.37^§^
Acute lower respiratory infection	23	961 (412–2073)	41 (22–80)	7 (2–14)	-
Pneumonia	17	280 (165–1006)	20 (6–70)	7 (2–9)	-
Others^†^	7	194 (117–469)	15 (8–209)	8 (2–22)	-
**Geographic location**	**-**	**-**	**-**	**-**	**0.37^§^**
Northern China	29	672 (302–961)	52 (21–78)	7 (3–9)	**-**
Southern China	50	1027 (267–2077)	30 (14–85)	4 (2–11)	**-**

Data are presented as median and interquartile range (IQR).

* One data set did not explain the diagnostic methods used. Others included culture (***n*** = 1), serological test (***n*** = 1), and not specified (***n*** = 3).

^†^ Others included pneumonia & bronchiolitis (***n*** = 1), severe acute respiratory illness (***n*** = 1), bronchiolitis (***n*** = 2), not specified (***n*** = 3).

^‡^ Because the three age groups overlapped with each other, statistical test was not performed to test the difference among them.

^§^ Values are for children aged ≤ 18 years because only this age group has sufficient data to allow a well powered analysis.

### Pooled estimates of per cent influenza-positive

The overall pooled estimates of the per cent influenza-positive among paediatric respiratory inpatients was 4.7% (95% confidence interval [CI]: 4.0–5.4%), 7.3% (95% CI: 6.4–8.1%); and 7.9% (95% CI: 7.1–8.7%) among children aged < 2 years, < 5 years and < 18 years respectively (**Table 3**). Considering the observed low sensitivity of immunoassay tests and the low specificity of APAAP tests, we did three additional analyses that excluded either one or both of them. However, children aged < 5 years and < 18 years consistently had higher point pooled per cent influenza-positive than children aged < 2 years. The 95% CIs of pooled per cent influenza-positive for children aged < 5 years and < 18 years overlapped considerably (**Table 3**).

**Table 3:** Pooled estimates of per cent influenza-positive of influenza-associated paediatric respiratory hospitalizations, by age group and by diagnostic test method in China, 1996–2012

-	Children aged < 2 years		Children aged < 5 years		Children aged < 18 years	
-	Number of data sets	Pooled per cent influenza-positive (95% CI)	Number of data sets	Pooled per cent influenza-positive (95% CI)	Number of data sets	Pooled per cent influenza-positive (95% CI)
Overall pooled per cent positivity	46	4.7 (4.0–5.4)	50	7.3 (6.4–8.1)	77	7.9 (7.1–8.7)
Northern China*	18	7.1 (5.3–8.9)	19	10.4 (8.3–12.4)	27	9.8 (8.2–11.5)
Southern China	28	3.8 (3.1–4.6)	31	5.9 (5.0–6.9)	48	7.1 (6.1–8.1)
Pooled per cent influenza-positive excluding immunoassay	24	6.9 (5.5–8.2)	28	10.0 (8.4–11.7)	45	10.5 (8.9–12)
Northern China	11	8.9 (5.7–12.1)	11	12.2 (8.4–16.1)	18	8.9 (10.5–12)
Southern China	13	6.0 (4.3–7.6)	17	9.0 (7.1–11)	27	8.9 (11.4–13.9)
Pooled per cent influenza-positive excluding APAAP	42	4.4 (3.7–5.1)	46	7.1 (6.2–8.0)	70	6.7 (6–7.4)
Northern China	17	6.7 (4.9–8.5)	18	10 (7.9–12.1)	27	9.5 (7.9–11.2)
Southern China	25	3.5 (2.7–4.3)	28	5.8 (4.7–6.8)	43	5.5 (4.6–6.3)
Pooled per cent influenza-positive excluding APAAP & immunoassay	20	6.6 (5–8.2)	24	10.4 (8.4–12.4)	38	8.8 (7.5–10.1)
Northern China	10	8.1 (5–11.3)	10	11.7 (7.7–15.7)	17	9.0 (6.7–11.4)
Southern China	10	5.7 (3.6–7.7)	14	9.8 (7.1–12.5)	21	9.3 (7.1–11.4)
Overall pooled per cent influenza-positive by PCR	5	7 (4.2–9.8)	9	8.9 (6.8–11)	13	8.8 (7.0–10.7)

*Northern China and Southern China are defined by national standards, which is Qing Mountain and Huai River line. (*15*)

In all age groups, per cent influenza-positive in the northern provinces was higher than that in the southern provinces (7.1% vs 3.8%, 10.4 vs 5.9%, 9.8% vs 7.1%). Additional stratified analyses by diagnostic test did not significantly change the pattern. The final pooled analyses of only PCR-confirmed data included 13 data sets; most of them were in the more developed eastern or southern provinces (*n* = 10 of 13). The point per cent influenza-positive remained higher among children aged < 5 years and < 18 years, but the 95% CIs of per cent influenza-positive of the three groups overlapped considerably (point per cent influenza-positive and 95% CI: 7% [4.2–9.8%] for < 2 years, 8.9% [6.8–11%] for < 5 years, and 8.8% [7.0–10.7%] for < 18 years).

## Discussion

Our study of influenza-associated severe hospitalizations from 23 provinces and autonomous administrative areas of China during the period 1996–2012 is the first systematic review of influenza-associated paediatric hospitalizations in China. Findings from this review complement results from China’s two influenza surveillance systems that are limited in their ability to capture the true number of influenza-associated paediatric hospitalizations either by using SARI as an overly specific case definition or by excluding many jurisdictions before 2012. Our review covered well developed provinces as well as the less-developed provinces for which only limited influenza-associated disease burden estimates are available. Using PCR-confirmed outcomes, we found that in addition to the significant burden of influenza in respiratory hospitalizations among children aged < 2 years, as observed in other northern hemisphere counties, the relative contribution of influenza was also high among acute respiratory hospitalizations in children aged < 5 years and < 18 years in China.

The fact that influenza is associated with severe outcomes among younger children as well as among older children is consistent with the SARI surveillance results from China and results from the systematic analysis on respiratory hospitalizations at the global level during similar study periods. (*13*, *14*, *28*) All three studies reported that influenza-associated hospitalization was higher among children of older age groups than among children aged < 2 years. Similar percentage of influenza-contributed respiratory hospitalizations among children < 18 years was also estimated from the global report (*14*) with 7.7% in developing countries and 8.5% in the WHO Western Pacific Region. Influenza not only contributes to respiratory hospitalization among children aged < 18 years, it also contributes to a significant percentage of outpatient visits. One study conducted in two northern provinces of China during 2012–2015 found that influenza was the most commonly detected virus in ambulatory patients across all age groups, (*29*) though this study used ARI for patient screening.

ARI was also the most commonly used case definition in all articles included in our analysis. This case definition is more sensitive than the strict SARI definition used in the surveillance system during 2011–2013. (*13*) In most populated developing country hospitals, including hospitals that conduct surveillance associated with severe outcomes of a respiratory virus, busy clinicians examining patients describe the patient’s general condition related with ARI rather than list numerous signs and symptoms in detail. (*30*) For future surveillance on influenza-associated severe outcomes, if clinicians are responsible for case enrolment or if enrolment is based on patient chart review, standardization and simplification of the case definition are encouraged to improve case capture and surveillance quality.

There was a substantial difference in the percentage by diagnostic test, with high positivity in those tested with immunoassay and low positivity in those tested by APAAP assay. Though studies have shown that PCR is more sensitive than other test methods, (*31*, *32*) our pooled results from other testing methods (not including APAAP and immunoassay) had higher positivity than PCR. This may be because the use of PCR was largely limited to the resourceful southern provinces that have lower proportions of influenza-associated hospitalizations compared with the northern provinces. Other testing methods were used with similar frequency among studies from southern and northern provinces.

We also found that the proportion of hospitalizations due to influenza was higher in the northern provinces than the southern provinces. As there were relatively fewer studies in Northern China, we suggest the strengthening of respiratory disease-related surveillance in Northern China to better understand the drivers of the disparity comparing with Southern China (e.g. etiologies, interventions, health seeking behaviours, influenza vaccine and pneumococcal vaccine uptake) to inform local prevention and control strategies.

Our analysis is subject to several limitations. First, the data sets were all from cities (prefectures) or referral hospitals in the provinces. Respiratory disease burden may differ between urban and rural areas, and we may not have adequately captured data from rural populations because of their limited access to city hospitals. Second, many data sets did not use PCR as a diagnostic test, particularly among northern provinces, raising uncertainty regarding the accuracy of their results. Although we attempted to address this limitation by generating a pooled estimate restricted only to PCR-based results, the pooled estimate is less representative of Northern China. Third, although we screened studies for use of clear criteria for influenza testing, it is possible that subjective clinical judgment may have influenced clinician testing practices and therefore our outcomes. Finally, we were not able to exclude 2009 influenza A(H1N1) data from nine data sets because the results were not stratified to allow this separate analysis.

Our study results suggest that influenza was responsible for almost 9% of paediatric respiratory hospitalizations. Though more studies are warranted on the influenza-associated outpatient burdens among these age groups and in Northern China, inclusion of school-aged children in the influenza vaccination priority group and collaborations with other organizations (for instance schools) to improve vaccine uptake may reduce substantial influenza-associated morbidities among children in China.
